# On the representativeness and stability of a set of EFMs

**DOI:** 10.1093/bioinformatics/btad356

**Published:** 2023-05-30

**Authors:** Francisco Guil, José F Hidalgo, José M García

**Affiliations:** Grupo de Arquitectura y Computación Paralela, Departamento de Ingeniería y Tecnología de Computadores, Facultad de Informática, Universidad de Murcia, Campus de Espinardo, Murcia 30100, Spain; Grupo de Arquitectura y Computación Paralela, Departamento de Ingeniería y Tecnología de Computadores, Facultad de Informática, Universidad de Murcia, Campus de Espinardo, Murcia 30100, Spain; Grupo de Arquitectura y Computación Paralela, Departamento de Ingeniería y Tecnología de Computadores, Facultad de Informática, Universidad de Murcia, Campus de Espinardo, Murcia 30100, Spain

## Abstract

**Motivation:**

Elementary flux modes are a well-known tool for analyzing metabolic networks. The whole set of elementary flux modes (EFMs) cannot be computed in most genome-scale networks due to their large cardinality. Therefore, different methods have been proposed to compute a smaller subset of EFMs that can be used for studying the structure of the network. These latter methods pose the problem of studying the representativeness of the calculated subset. In this article, we present a methodology to tackle this problem.

**Results:**

We have introduced the concept of stability for a particular network parameter and its relation to the representativeness of the EFM extraction method studied. We have also defined several metrics to study and compare the EFM biases. We have applied these techniques to compare the relative behavior of previously proposed methods in two case studies. Furthermore, we have presented a new method for the EFM computation (PiEFM), which is more stable (less biased) than previous ones, has suitable representativeness measures, and exhibits better variability in the extracted EFMs.

**Availability and implementation:**

Software and additional material are freely available at https://github.com/biogacop/PiEFM.

## 1 Introduction

Cellular metabolism has essential applications in biology and medicine, such as the analysis of different types of cancer and other diseases (Bazzani 2014) or in biotechnology (Jang *et al.* 2012, Woo and Park 2014). Metabolism can be thought of as a network of reactions transforming metabolites into other metabolites. A possible way of studying cellular metabolism is by analyzing the structural properties of this network. This study is usually performed using graph-related techniques or constraint-based modeling. The latter term was introduced by Covert and Palsson (2003) and, in this approach, the network is modeled as a hypergraph with several constraints restricting the set of possible fluxes. These constraints can be stoichiometric (based on the quantities of metabolites involved in each reaction), thermodynamic (limiting the direction of some reactions), or regulatory (generally based on gene regulations of the network).

An admissible flux distribution (called a mode or a pathway) is defined as a set of reactions where all intermediate compounds are balanced and irreversible reactions run in the appropriate direction (Klamt and Stelling 2003). For any nontrivial network (i.e. a network that can have more than one possible non-zero mode), its set of modes is infinite, so the focus is on finding finite subsets that could generate the complete set of modes. Two of the most used subsets are the so-called elementary modes (Schuster and Hilgetag 1994) and the extreme pathways (Klamt and Stelling 2003), and both definitions agree when all the network’s reactions are irreversible (Klamt and Stelling 2003). If this is not the case, each reversible reaction can be modeled using two irreversible ones (Gagneur and Klamt 2004). This work assumes that all reactions are irreversible and uses the term elementary mode (referred to as elementary flux mode or EFM) to be minimal or extreme. Even though the number of different EFMs has an upper bound (Klamt and Stelling 2002), this number is usually quite large.

Several strategies have been proposed to calculate the set of EFMs of a network. They can be divided into non-biased methods and biased ones. The first ones try to find the whole set of EFMs by using techniques related to the double description method (DDM) (Fukuda and Prodon 1996) or pivotal ones (Avis and Fukuda 1992). Both methods are computationally demanding in terms of time and memory required: the first in terms of memory usage while the second is CPU limited with marginal memory requirements, so they can only be used in small- to medium-sized networks. A relatively complete list of non-biased methods can be consulted in Ullah *et al.* (2020).

The cardinality of the set of EFMs is extremely large for genome-scale networks, and this central problem can be faced with algorithms that only compute smaller subsets of EFMs. Trying to infer properties of the networks from the computed subset introduces potential biases. These (potentially) biased methods are less expensive and are usually based on posing successive optimization problems to obtain a subset of the set of EFMs. Especially those based on linear optimization (LP) techniques are suitable in constructing EFMs because these methods are computationally efficient, and many available libraries can be used. One of the main difficulties that arise from using biased methods is the occurrence of repeated EFMs. The relative efficiency of a technique is usually measured in terms of time needed to obtain a certain number of different EFMs or in the ratio between the number of EFMs obtained and the number of optimization problems solved (Pey *et al.* 2015, Guil *et al.* 2020b).

The main problem is the representativeness of the subset of EFMs computed by biased methods (LP methods precisely). This representativeness can be naively understood as the similarity between the EFMs obtained and those not calculated. It is commonly accepted (Machado *et al.* 2012, Tabe-Bordbar and Marashi 2013) that two key measures to study this representativeness are the mean length of the supports of the EFMs and, for each reaction of the network, the proportion of EFMs in which the reaction appears as active. Using them as primary measures to compare the quality of different methods seems natural.

The main goal of this article is to develop a unified framework to study the quality of the EFM extracting methods, including their representativeness. As far as we know, this is the first appearance in the literature of this kind of study, and we apply it to compare several previously proposed methods. We also developed a new way [called Pi(votal)EFM] that can be viewed as a mixture of biased (LP) and non-biased (pivotal) methods that overcome some of the difficulties associated with biased and non-biased methods.

The main contributions of this article are the following:

- Definition of quality measures.
- Description and implementation of a new method to compute sets of EFMs that overcome most of the issues associated with previously proposed methods.
- Analysis of previous methods and comparison with the newly proposed one.

The rest of the article is arranged as follows. Section 2 provides definitions for biological networks and background to elementary modes, reviewing previously proposed methods and recalling results and techniques from linear programming. Section 3 presents the proposed quality indicators and explains our algorithm for finding EFMs. Section 4 is devoted to applying our new method to two different case studies and comparing its relative quality with previously proposed ones. The article ends with some conclusions.

## 2 Background

A metabolic network *N* is a tuple (*M*, *R*, *S*) where *M* is a set of (internal) metabolites, *R* a set of reactions, and *S* a stoichiometric matrix $S\in R^{M\times R}$. Each row represents a metabolite in this matrix *S* and the value in each matrix entry is the stoichiometric coefficients for metabolites on the corresponding reaction.

A vector is defined for any possible state of the network. This vector represents the rate at which the substrate metabolites are converted to product metabolites by a given reaction. The vector that contains the reaction rates is called the flux rate.

A vector *v* representing flux rates of the reactions is called a mode if it fulfills the steady-state and thermodynamic constraints: where the last condition stands for v[i]≥0 for all the components v[i] of *v* (recall that all reactions are assumed to be irreversible).


(1) S·v=0

(2) v≥0,

### 2.1 Computing EFMs

The cone of solutions *C* of the network is defined as the set of all the modes of the networ*k*


$$C=\{v\in R^{R}\;|\;S\cdot v=0,\;v\geq 0\}.$$

For any mode *v*, its support, supp(v), is the set formed by those reactions *r* that appear with a non-zero rate in *v*. We will assume that all reactions are non-blocked (see Burgard *et al.* 2004) that is, for each reaction r∈R there is at least a mode *v* such that *r* is active in *v*. A mode *v* is called an elementary mode (EFM) if there is no other non-zero mode v′ with supp(v′) ⊊ supp(v) (Schuster and Hilgetag 1994). It is well known that the set of EFMs is finite in any network, and any mode can be written as a sum of non-negative multiples of EFMs (see Gagneur and Klamt 2004).

Theorem 1 [see the demonstration in Klamt *et al.* (2005) or Terzer and Stelling (2008)] gives an algebraic characterization of EFMs in terms of the stoichiometric matrix *S.*

Theorem 1 *Given a mode v, consider the submatrix S_v_ of S including those columns corresponding to reactions in supp(v) and rows associated with metabolites that appear in those reactions. The mode v is an EFM if and only if* $\mathrm{rank}(S_{v})=k-1$*for* k=|supp(v)|.

As observed previously, we can distinguish between non-biased and biased methods. Non-biased methods try to find the whole set of EFMs and most of them are based on the DDM (Fukuda and Prodon 1996) or the pivotal (also known as lexicographic reverse search) method (Avis and Fukuda 1992). On the other hand, biased methods differ from non-biased ones in that they only try to compute subsets of the set of EFMs. They are usually faster than non-biased methods and can provide EFMs for any network. Several approaches have been proposed so far using a wide variety of techniques. Inside these methods, it is possible to find modifications of the DDM to limit the number of intermediate steps to overcome the memory and time limitations of DDM (Machado *et al.* 2012). Other developments have been explored, such as methods that rely on graph theory concepts (usually only applicable to small networks; Arabzadeh *et al.* 2018), methods based on discarding non-feasible EFMs (see Gerstl *et al.* 2015, Jungreuthmayer *et al.* 2015), or methods that reduce the considered network (Kaleta *et al.* 2009, Marashi *et al.* 2012).

The most frequent technique used in biased methods is optimization. In these approaches, the steady-state and thermodynamic constraints define the starting point of the algorithm. By defining an *ad hoc* function and imposing additional constraints, optimization problems are defined whose solutions are EFMs of the network. Varying this function and the additional constraints enable us to pose different problems and, in principle, obtain different EFMs.

Inside this family, there are methods using mixed-integer linear optimization (MILP) methods as in the well-known *k*-shortest algorithm (De Figueiredo *et al.* 2009). Other methods rely on linear optimization (LP) methods as CASOP (Bohl *et al.* 2010), the one proposed by Marashi *et al.* (2012) (Tabe-Bordbar and Marashi 2013), treeEFM (Pey *et al.* 2015), FLFS-FC (Guil *et al.* 2020a), or EFM-Ta (Guil *et al.* 2020b). Finally, some methods use a hybrid approach between MILP and LP as alternate integer linear programming (AILP) (Song *et al.* 2017).

A well-known issue in most optimization-based methods is the appearance of repeated EFMs. This issue is associated with LP optimization methods and does not occur in MILP-based ones. These repeated EFMs appear because posing different optimization problems does not guarantee that the corresponding EFMs are also different. Different strategies can be used to ensure a variety of computed EFMs: deleting random reactions (Tabe-Bordbar and Marashi 2013), blocking reactions from the last EFM obtained (Bohl *et al.* 2010), doing so recursively by using trees (Pey *et al.* 2015), or blocking those reactions that appear in those EFMs with a high rate of repetitions (Guil *et al.* 2020a). By additionally restricting the number of steps performed by the optimization method (Guil *et al.* 2020b), reasonable efficiency rates can be achieved even in large networks, measured in terms of time or as the quotient between the number of optimization problems solved and the number of EFMs obtained (see Pey *et al.* 2015, Guil *et al.* 2020b).

Another problem that arise while working with biased methods is the representativeness of the set of EFMs obtained. Since the complete set of EFMs is not available, the study of metabolic properties only can be done in function of the computed ones that can present a significant bias. This issue has been previously observed (see Machado *et al.* 2012, Tabe-Bordbar and Marashi 2013, Ullah *et al.* 2020) and is a severe limitation that affects the usability of these methods. Note that different methods provide subsets that can be different in a statistically significant way (Hidalgo *et al.* 2018), so properties inferred from these EFMs may heavily depend on the method used.

### 2.2 Optimization techniques for EFMs

The cone *C* of solutions of Equations (1) and (2) can be converted into an optimization problem by introducing any function *f* on the variables representing the fluxes through the reactions. This function can be maximized or minimized to get specific points of interest in *C*.

The associated optimization problem can be stated a*s*


(3) Optimize  f=∑i=1naiv[i]subject to S·v=0 v[i]≥0 ∀ri∈R.

Notice that in problem (3), the decision variables are the {v[i]} and the function coefficients $\left\{a_{i}\right\}$ can be thought of as parameters that allow us to pose different LP problems. We call this optimization problem a clean LP problem.

Maximizing these functions can produce non-bounded problems, while minimizing them usually produces trivial solutions. A common way to overcome these issues is the introduction of additional constraints in the variables.

Given two sets of reactions $T_{1},T_{2}\subset R$, the restriction*s* are called the positive and negative constraints associated with *T*_1_ and *T*_2_. These reaction sets are sometimes called positive (or negative) seeds (see Acuña *et al.* 2009, Hidalgo *et al.* 2016).


(4) $$\sum_{i\in T_{1}}v[i]=1$$

(5) $$\sum_{i\in T_{2}}v[i]=0$$

Adding a positive restriction (4) to the clean LP problem ensures that any solution obtained contains at least one reaction from *T*_1_ [or the absence from the solution of all the reactions in *T*_2_ for the negative restriction (5)] so trivial solutions are avoided while minimizing functions. Observe that two or more negative constraints can be joined into a single constraint, but this is not true for positive ones.

The following theorem (see Pey and Planes 2014) shows that, when restricting the number of additional positive restrictions to one, any solution obtained by solving a feasible LP problem is an EFM of the networ*k*.

Theorem 2 *Given any non-empty subset* T⊂R*and a non-zero vector* ${\left(a_{i}\right)}_{1}^{n}\in R^{n}$*with* $a_{i}\geq 0$*, consider the LP problem obtained by adding to the clean LP problem the positive constraint associated with T. This problem is feasible and the solution obtained is an EFM of the network*.

*Let* T′⊂R*be another subset of reactions. If we impose both the positive and negative constraints associated with T and* T′*, the problem is unfeasible or the solution obtained is an EFM.*

## 3 Methods

For a proposed EFMs extraction algorithm, we propose the following metrics to measure its quality:

- Efficiency: the efficiency rate at which we can compute new EFMs. At least for algorithms based on LP methods, it is better (see Pey *et al.* 2015, Guil *et al.* 2020b) to measure the efficiency in terms of the number of EFMs computed by some LP problems solved and not in terms of time required. Clearly, in most cases, these two possible measures are closely correlated.
- Proportion of EFMs calculated: at least for small- to medium-size networks, the method should be able to compute a high percentage of the EFMs of that network.
- Statistical meaning: the method should provide good approximations for parameters such as the mean length of EFMs computed or the proportion of EFMs containing each reaction *r*, defined as

$p(r)=\frac{\left|E\prime \in E\;|\;r\in \mathrm{supp}(E\prime )\right|}{\left|E\right|}$ _**,**_

where *E* stands for the whole set of EFMs.

Two important questions associated with the biological representativeness of a set of EFMs computed by any method of extraction are the following:

- Detect hidden biases, i.e. identify possible biases associated with this method and correct them (if possible).
- Answer the critical question, “when can we stop an extraction process?”. That is, at which point, continuing with our process will not provide a better understanding of the network.

As far as we know, the unique indicator that has been used to compare different methods has been the efficiency one. As we show in Examples 1 and 2, this can be misleading because the obtained subset E′ can be very different from *E*. Depending on the problem we want to address, it is preferable to use several (or all) the proposed indicators.

### 3.1 Representativeness and parameter stability

Let denote by *E* the whole set of all the EFMs from a network *N* and let E′⊂E be a subset. A subset E′ is representative of the set *E* (for a specific parameter *λ*) if the measure obtained from *λ* in E′ is similar to that obtained in *E*.

We can define different parameters in *E*. We distinguish between two classes of parameters:

- Reaction agnostic parameters: it is a characteristic of the elements in *E* that does not consider the specific reactions that appear in the (support) of the EFMs in *E*. The primary example is the mean length of those EFMs.
- Reaction-based parameters: those parameters computed in terms of the reactions in those EFMs. The proportion of EFMs containing a specific reaction *r*, *p*(*r*), is a reaction-based parameter.

Even if the set *E* can be computed, studying the representativeness of E′ concerning a specific parameter is difficult.

Example 1 *We use the core Escherichia coli model (Orth et al. 2010) as a case study.*

*A well-known method to compute EFMs using MILP techniques is the k-shortest algorithm that can compute all the EFMs in increasing length order. Throughout this article, the implementation of k-shortest included in CobAmp is used* (Vieira and Rocha 2019)*. Suppose we implement another method called k-largest that computes the same EFMs in decreasing length order (we simulate this method by ordering the set of EFMs by decreasing length).*

*We apply both methods and compute two subsets* E′,E″⊂E*, each containing 5000 EFMs. The subsets* E′*and* E″*are different, so we can ask which one is more representative of E about the global parameter “length” or the local parameters* ′p(r)′*for each reaction r.*

*Regarding the reaction agnostic property “length,” the actual mean length of all the EFMs E is 48.1163. The mean length in the subsets* E′*and* E″*are 33.886 and 53.6386. As expected, none of those estimations are a good approximation of the actual mean.*

**Figure 1.**
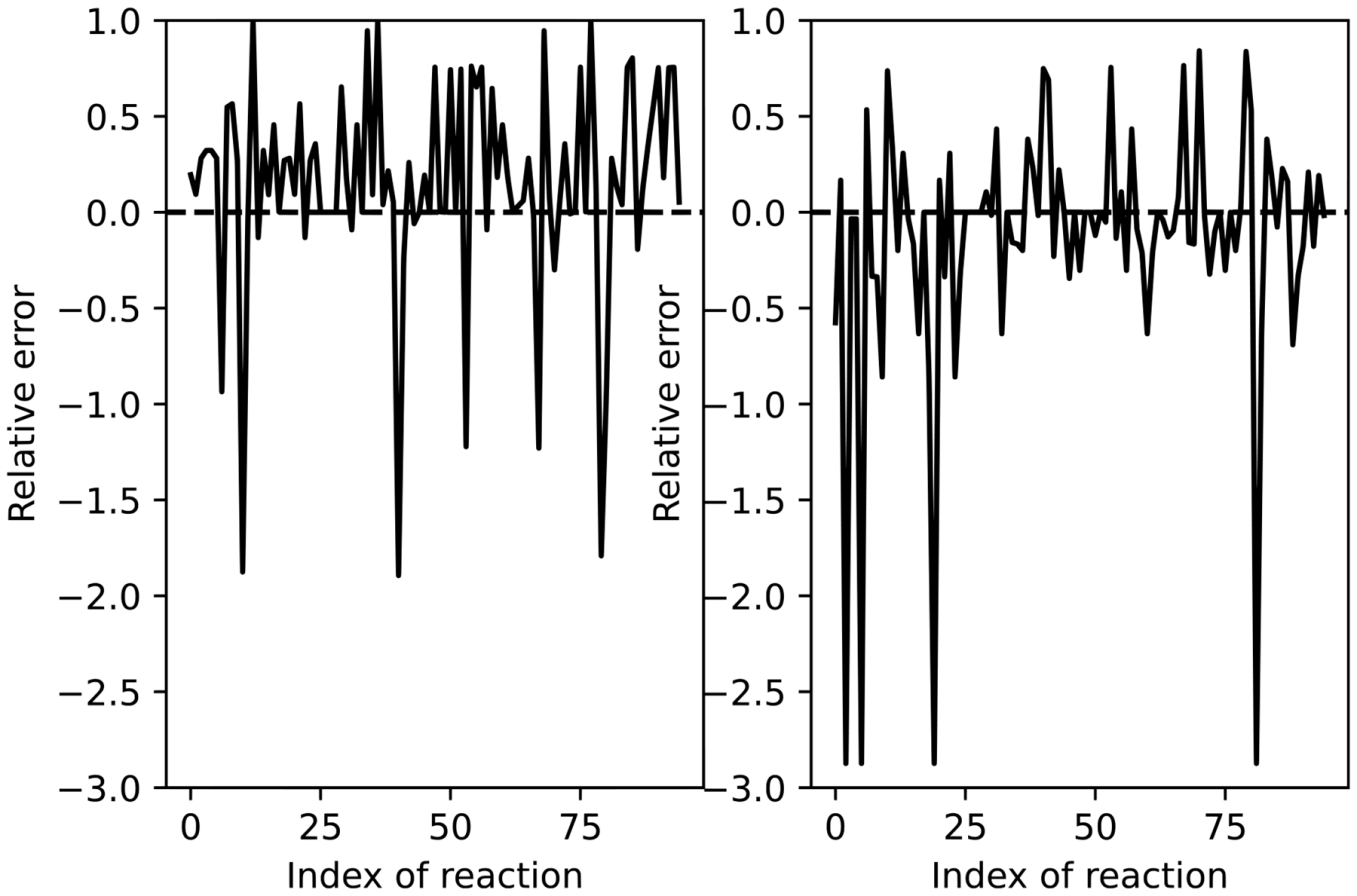
Relative errors for the proportion of EFMs containing each reaction by using the EFMs computed using the *k*-shortest method (left) and the simulated *K*-largest one (right).

*If we study the proportions p(r) obtained for all reactions r in N, the results vary depending on the chosen reaction. Figure 1 shows the relative errors that appear while trying to approximate the proportions p(r) in E by those computed in* E′ *and* E″**(***all relative errors computed through this article are always measured in absolute value, i.e. the relative error obtained while approximating a by another value* a′*is* $\frac{\left|a-a\prime \right|}{\left|a\right|})$.

*Most errors in the second algorithm are concentrated in four reactions, but it turns out that these reactions are, in fact, partially coupled {i.e. for any pair* $\left\{r_{i},r_{j}\right\}$*among them and for any EFM E^k^ of the network, r_i_ is in the support of E^k^ if and only if r_j_ belongs to it [see Burgard et al. (2004) or Larhlimi et al. (2012)]}. The same assertion is invalid when considering the six reactions that mainly produce errors in the first algorithm.*

**Figure 2.**
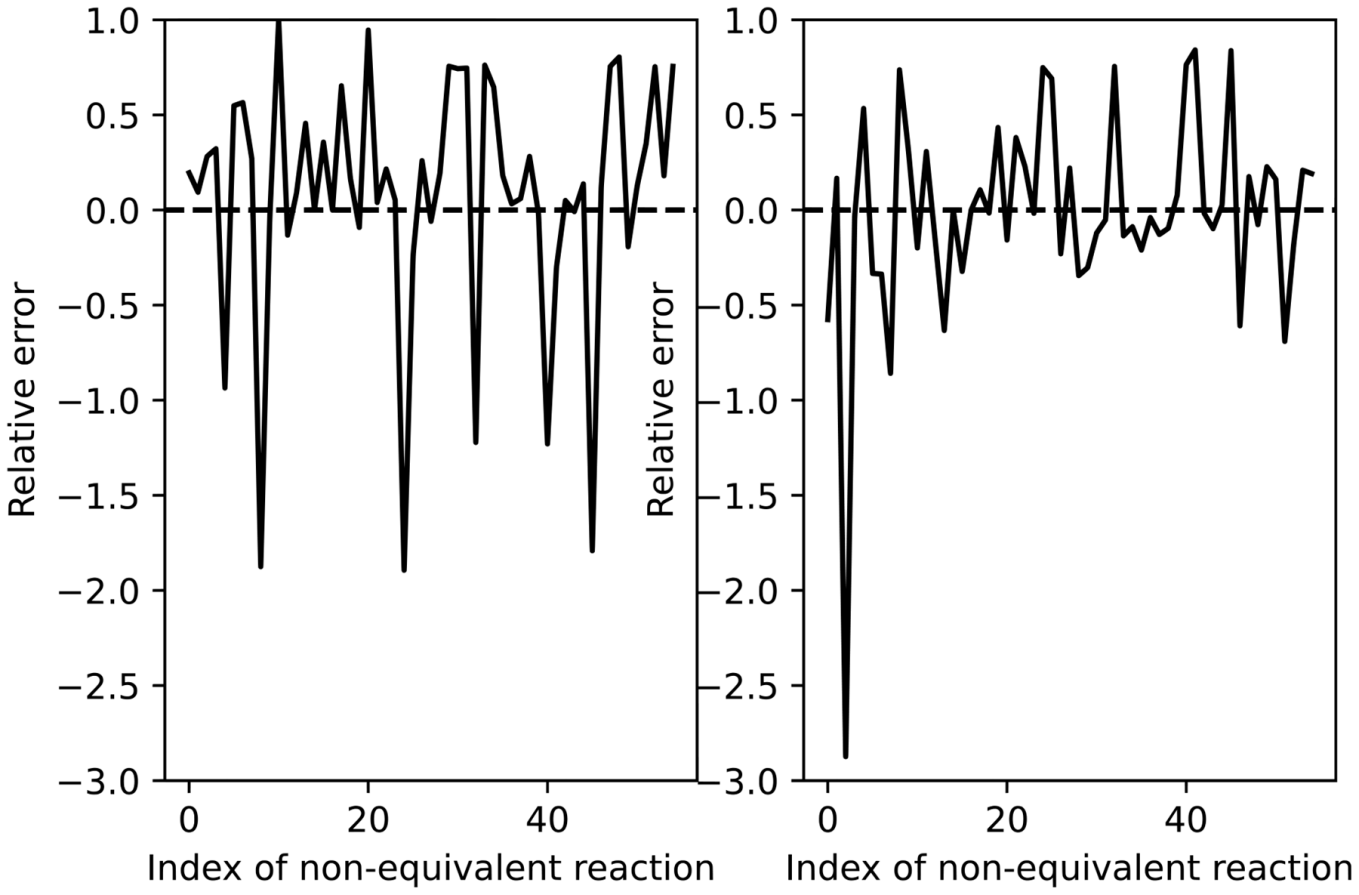
Relative errors for the proportion of EFMs containing each non-partially coupled reaction by using the EFMs computed using the *k*-shortest method (left) and the simulated *K*-largest one (right).

*If we reproduce the study but only consider non-partially coupled reactions, Fig. 2 looks quite different.*

*A primary statistical study shows that the first method initially produces a mean relative error of 0.4255 (with a standard deviation of 0.4222), which is slightly worse than the one associated with the second method which is 0.4072 (with a standard deviation of 0.5880). After correcting the effect produced by partially coupled reactions, we get mean relative errors of 0.1940 and 0.1175 (with standard deviations of 0.2258 and 0.1061, respectively). This result is consistent with that few EFMs in core E.coli of small length, but most have lengths near 53 (those obtained with the second method).*

So, even in the few cases in which the whole set *E* can be computed, it is pretty easy to obtain two subsets of EFMs that are quite different (see Hidalgo *et al.* 2018), but it is much harder to elucidate which one is more representative.

A different approach consists of trying to answer the reverse question: when can we be sure that a given set E′ cannot represent *E*? More precisely, we want to obtain a criterion for a method of extraction of EFMs that assures us that the associated subset of obtained EFMs cannot be representative.

To do so, we can compare the method with itself. Suppose we have an algorithm *A* that produces EFMs and denote by $E\prime_{n}\subset E$ the subset formed by the first *n* EFMs obtained by this method. Let us take two different (and large enough) values *n*_0_ and *n*_1_, and compute two subsets $E\prime_{n_{0}},E\prime_{n_{1}}\subset E$. If our method is representative for a parameter *λ*, then the parameters $\lambda_{n_{0}}$ and $\lambda_{n_{1}}$ obtained in the subsets $E\prime_{n_{0}}$ and $E_{n_{1}}$ must be also similar. We call this condition *stability for the parameter* *λin the algorithm A*.

Observe that representativeness implies stability, but the converse is not valid.

**Figure 3.**
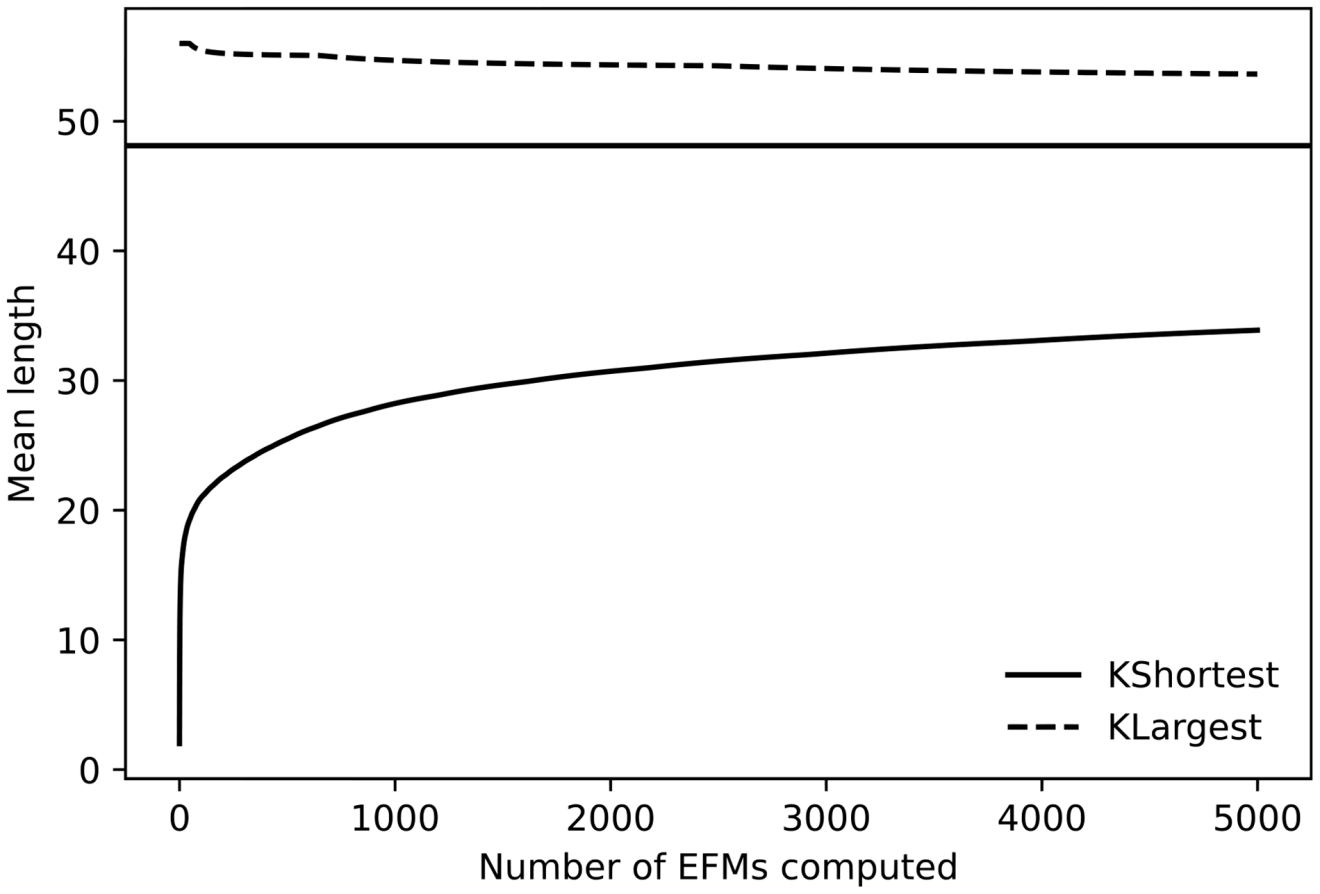
Evolution of the mean length of the EFMs obtained using the *k*-shortest method (above) and the simulated *K*-largest one.

Example 2 *We return to Example 1. Figure 3 shows the evolution of the parameter “mean length” using both methods.*

*In this case, method 1 is less stable (and representative) for this parameter than method 2 (we have added a horizontal line indicating the correct parameter value in this network).*

*Let us take as reaction r the production of Biomass and study the evolution of the parameter p(r). The first method has always produced EFMs that do not contain the Biomass reaction, while the second one has produced EFMs that always contain it. So, the parameter p(r) is completely stable for both methods, but the obtained approximations are not representative.*

*In this case, the stability of the parameter is the same for both methods, but method 2 is still more representative than method 1 [the correct value of p(r) is closer to 1].*

Let us return to the questions posed at the beginning of this section,

- Detect hidden biases: Figure 3 shows an expected effect; method 1 tends to produce shorter EFMs while method 2 produces large ones. The other bias was unclear: the behavior of both methods is different regarding the Biomass reaction and is highly biased.
- When can we stop an extraction process? A partial answer is that the extraction process must continue while any parameter is unstable. As observed, the extraction process can stop without having a representative subset E′ yet. However, in this case, stability tells us that continuing the process will likely produce “similar” EFMs. It probably will produce larger subsets but no more representative ones (at least if the chosen parameters reflect the question of interest). If the results are inconsistent, a much larger set must be obtained or we should switch to another method.

As seen, some biased methods tend to produce EFMs that are all too similar. To test the representativeness of the extracted EFMs, we propose to use the number of pairs of compatible reactions (i.e. pairs of reactions such that there is, at least, an EFM containing both in its support) as a measure of the variability of the obtained set of EFMs. So, a small number of such pairs may indicate a highly biased method. In this article, we use a simplified version of this metric that accounts for the number of reactions that are compatible with the corresponding Biomass reaction.

The representativeness study can only be carried out in networks where we can obtain the whole set of EFMs. In these networks, biased methods are unnecessary (although they can be used for efficiency purposes). On the other hand, studies on the stability of the parameters can be performed in any network and inform us of the biases produced by any EFM extraction method we use.

### 3.2 The Pi-EFM method

In this section, we introduce a new EFM extraction method called PiEFM. This method is a mixture of LP (biased) and pivotal (non-biased) methods. This method shows good behavior relative to our proposed quality measures (see Section 4).

Let us start with a network in which all reactions are non-blocked and irreversible. To do so, all blocked reactions are deleted and any reversible reaction is decoupled into two new reactions representing the possible directions (Guil *et al.* 2020a). Reactions that do not come from this decoupling process are called (originally) irreversible.

Let us start with any LP method to compute a small number of EFMs. A highly biased set of EFMs would be found if starting with a purely random method because this procedure tends to obtain shorter ones. On the other hand, always choosing the same target reaction *r* and adding the additional positive restriction *r*** **=** **1 also induce an initial bias because we would not allow the obtention of EFMs not including it. These kinds of biases could be partially avoided by following this easy algorithm:

- For each reaction *r*, obtain at least an EFM containing it. To do so, add the additional restriction *r* = 1 and a randomly constructed function *f* and solve the associated LP problem given by formula (3) and the new positive constraint v[r]=1.
- Fix a number *n* as the total amount of LP problems that we are going to solve.
- For any attempt in the range {1,⋯,n}_**,**_ we add the additional positive restriction *r*_Biomass_ = 1 where *r*_Biomass_ stands for the (artificial) reactions that account for the biomass production. We also add a random function *f* constructed by selecting random positive coefficients *a_i_* to the variables.
- Solve the associated LP problem obtained by adding the positive constraint to the clean LP problem. If this EFM has not been previously computed, add it to the list of computed ones and keep records of the number of times each EFM has been computed.

This part of the method is biased. Observe that this list of initial EFMs can be computed just once and saved in memory so the results obtained from now on can be reproduced.

The second part of the method consists of extending this list by the following algorithm:

- In each step, choose a previously calculated EFM with the condition of having been computed the least number of times and not having been explored yet. Mark it as explored.
- For this EFM, E′, elaborate an optimization problem with E′ as optimal solution. To do so:
   – Select the set *I* of all reactions in supp(E′) that come from initially irreversible ones and consider the additional positive restriction $\sum_{I}^{}v[i]=1$.
   – Construct the function $f=\sum_{i\notin \mathrm{supp}(E\prime )}^{}v[i]=1$
   – Consider the associated LP problem
    Minimize f=∑i∉supp(E′)v[i]subject to S·v=0 ∑i∈Iv[i]=1 v[i]≥0 ∀ri∈R

Observe that this function has 0 as minimum value and that this minimum can only be attained at E′.

- As suggested in Guil *et al.* (2020b), from the final tableau of this optimization problem it is possible to perform additional steps to obtain new adjacent vertices of the cone *C*. To do so, start by retrieving the sets *J*_1_ and *J*_2_ containing those variables that are basic or non-basic in this solution. For each pair $(j_{1},j_{2})\in J_{1}\times J_{2}$, perform an additional pivoting step trying to replace the basic variable *j*_1_ with the non-basic one *j*_2_. If possible, the new solutions obtained are also a vertex of the cone *C* and so an EFMs of the network (see Pey and Planes 2014, Guil *et al.* 2020b).
- If new, these new EFMs are added to the list, and finally, the number of occurrences of each EFM is updated.
- Repeat this process until a certain stop criterion is fulfilled (maximum number of EFMs to be computed or time limit).

## 4 Case studies

Our evaluation platform has a double socket Cascade Lake Xeon Gold 6238 (44 cores) @ 2.2 GHz with 384 GB of RAM. The system runs on a CentOS Linux 7.5, running CPLEX, version 12.10 from IBM and Python 3.6.8 from Intel (https://www.ibm.com/academic/topic/data-science).

As case studies, we have chosen two different network models available from BIGG Models (Schellenberger *et al.* 2010). The first model considered is the educational model *core E.coli (*Orth *et al.* 2010), having 95 reactions and 72 metabolites. This model has been exhaustively studied and all its EFMs can be computed using, e.g. efmtool (Terzer and Stelling 2008). The second model is the reconstruction model for *E.coli* iAF 1260 (Feist *et al.* 2007) with 2382 reactions and 1668 metabolites. This model has been previously used in Pey *et al.* (2015). Both models have been imported using *COBRAPy (*Ebrahim *et al.* 2013).

Not all the previous EFM extraction methods are currently available, so we have limited our analysis to treeEFM (Pey *et al.* 2015), *k*-shortest EFM (De Figueiredo *et al.* 2009), emsampler (Machado *et al.* 2012), EFM-Ta (Guil *et al.* 2020b), FLFS-FC (Guil *et al.* 2020a), and our proposed PiEFM method.

In addition, we have also included a purely random method that is only used as a base test. This method consists of choosing, at each step, a random linear function in the reactions and imposing an additional positive constraint that is also random in order to pose an LP problem and compute an EFM of the network.

### 4.1 EFM extraction methods in *core E.coli*

There are precisely 100 274 EFMs in this network. Therefore, in this case, the exact value of its mean length and, for each reaction *r*, the proportion of EFMs containing it in its support, *p*(*r*), can be computed. As noticed, there is no need for biased methods in this case, but it offers an excellent example to test all the proposed methods using our representativeness measures.

We do not compare in detail the efficiency of each previously published method (the information is provided in the corresponding references). For completeness, we only include a brief study of the number of EFMs that can be computed for each method in a fixed time interval. Being a relatively small network, we have used a fixed time of 5 min for our study. The obtained results are displayed in Table 1.

**Table 1.** Number and proportion over total of *E.coli core* EFMs computed by different algorithms using a time limit of 5 min

Method	Computed EFMs	Percentage over total	Time elapsed (s)
*K*-Shortest	434	0.43	300
EFM-Ta	23015	22.95	300
FLFS-FC	10340	10.31	300
TreeEFM	69336	69.14	300
PiEFM	100274	100	145

Regarding RAM memory consumption, we must take into account that part of this memory is used to store the obtained EFMs. So, in this case, we have fixed the number of EFMs obtained to 10 000 and studied the RAM requirements. Results are summarized in Table 2.

**Table 2.** RAM consumption, in megabytes, while computing the first 10 000 EFMs by different algorithms

	EFM-Ta (MB)	FLFS-FC (MB)	TreeEFM (MB)	PiEFM (MB)
RAM used	219	328	253	199

Due to time limitations, *K*-shortest algorithm has not been included in this study. But, even for computing the first 500 EFMs, the RAM memory consumed by this method raised to 432 MB.

A more detailed study of the efficiency of the new proposed method PiEFM can be found in the Supplementary Material.

Instead, we will focus on the remaining metrics: the number of EFMs that can be computed and the statistical meaning of the obtained EFMs.

Let us start with the proportion of EFMs that can be computed. We must make some remarks:

- We have not included emsampler in the remaining analysis because it is not a sequential method; we cannot retrieve the EFMs in the order they are computed.
- To execute treeEFM, we need a target reaction present in all the computed EFMs. In order to avoid biases, we have chosen the reaction *GLCpts* that is active in 100 273 of the 100 274 EFMs of the network.
- We have computed 15 000 EFMs for each method imposing a time limit of 10 min. All methods have calculated this number of EFMs except the purely random method, which has computed only 4872 different ones.

**Figure 4.**
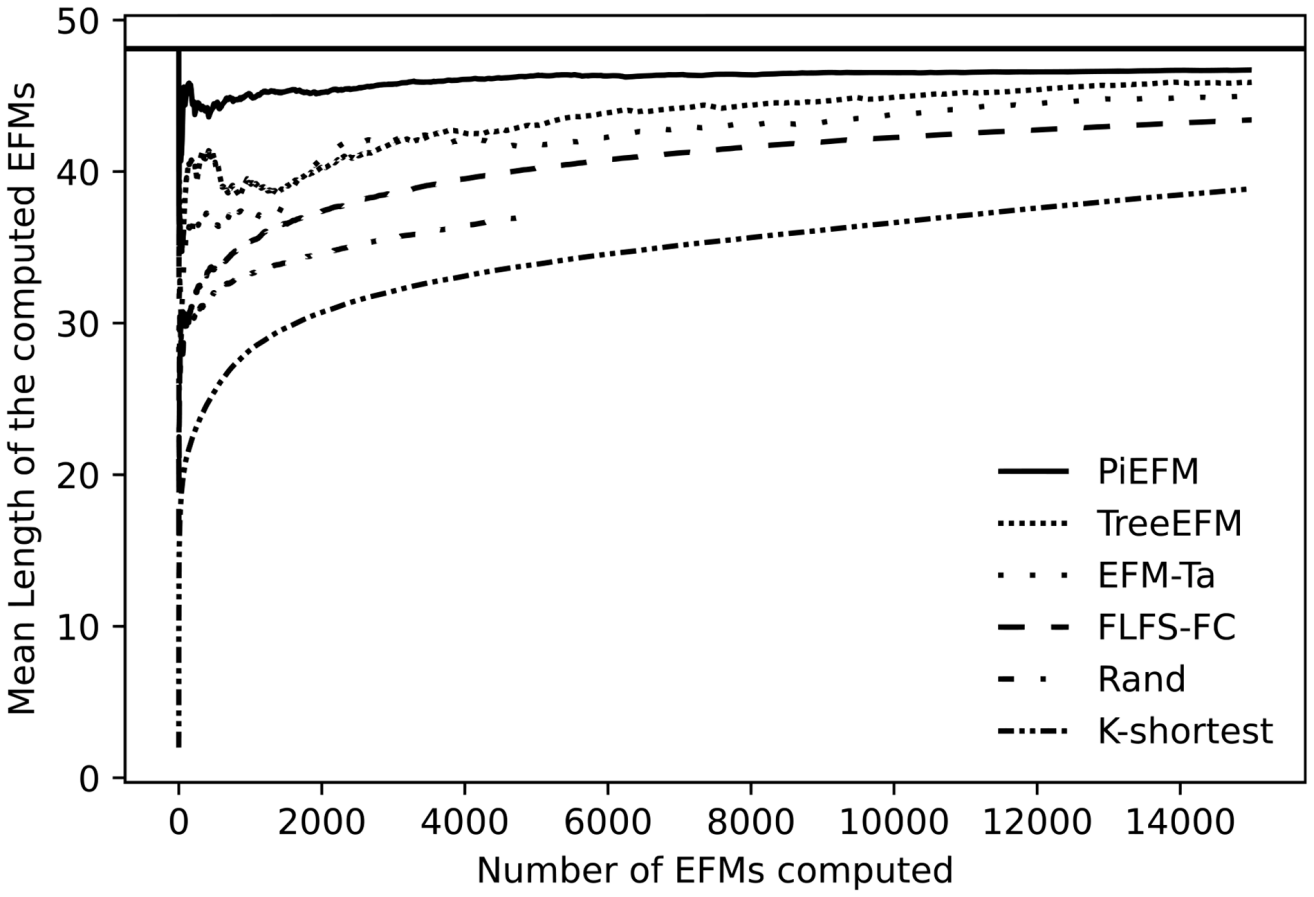
Evolution during the experiment of the mean length of the computed EFMs using different methods in *core E.coli*.

According to the mean length of the computed EFMs, there is a wide variation among the methods that can be observed in Fig. 4. As expected, the *k*-shortest method is biased for this metric and the same occurs with the random one. FLFS-FC, EFM-Ta, and treeEFM have an intermediate behavior and PiEFM provides excellent approximations of the correct value of this mean.

**Figure 5.**
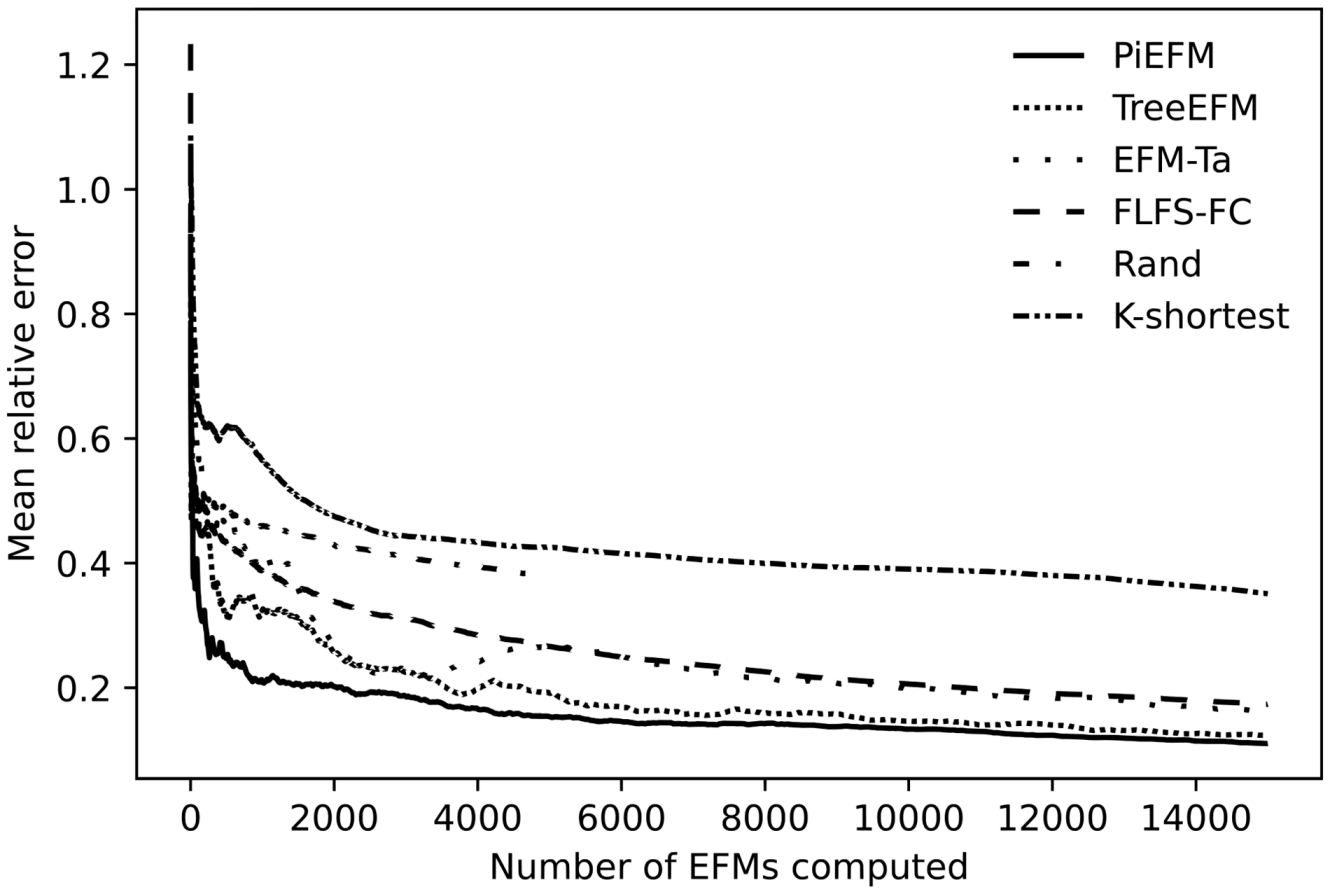
Evolution during the experiment of the mean relative error (right) between the real proportions of EFMs containing each reaction and those calculated on the computed EFMs using different methods in *core E.coli*.

For each reaction, *r*, the approximations of the parameters *p*(*r*) obtained from our methods can also be studied. A standard way to do so is by computing the Pearson correlation coefficient between the correct parameters *p*(*r*) and the approximated ones $p_{k}(r)$, where $p_{k}(r)$ stands for the proportion of EFMs containing the reaction *r* after extracting the first *k* EFMs (Machado *et al.* 2012). This difference can also be measured as the mean relative error between the values of *p*(*r*) and $p_{k}(r)$, where R′⊂R denotes the set of non-blocked reactions. Both measures are similar, but the mean relative error is the most sensitive one, so we will use it from now on. The evolution of the mean relative error is shown in Fig. 5.


$$\mathrm{mre}(p_{k},p)=\frac{\sum_{r\in R\prime }^{}\frac{\left|p_{k}(r)-p(r)\right|}{p(r)}}{\left|R\prime \right|},$$

Again Fig. 5 shows that *k*-shortest EFM and the random methods are both highly biased, EFM-TA and FLFS-FC present smaller biases, and treeEFM and PiEFM provide good approximations.

**Figure 6.**
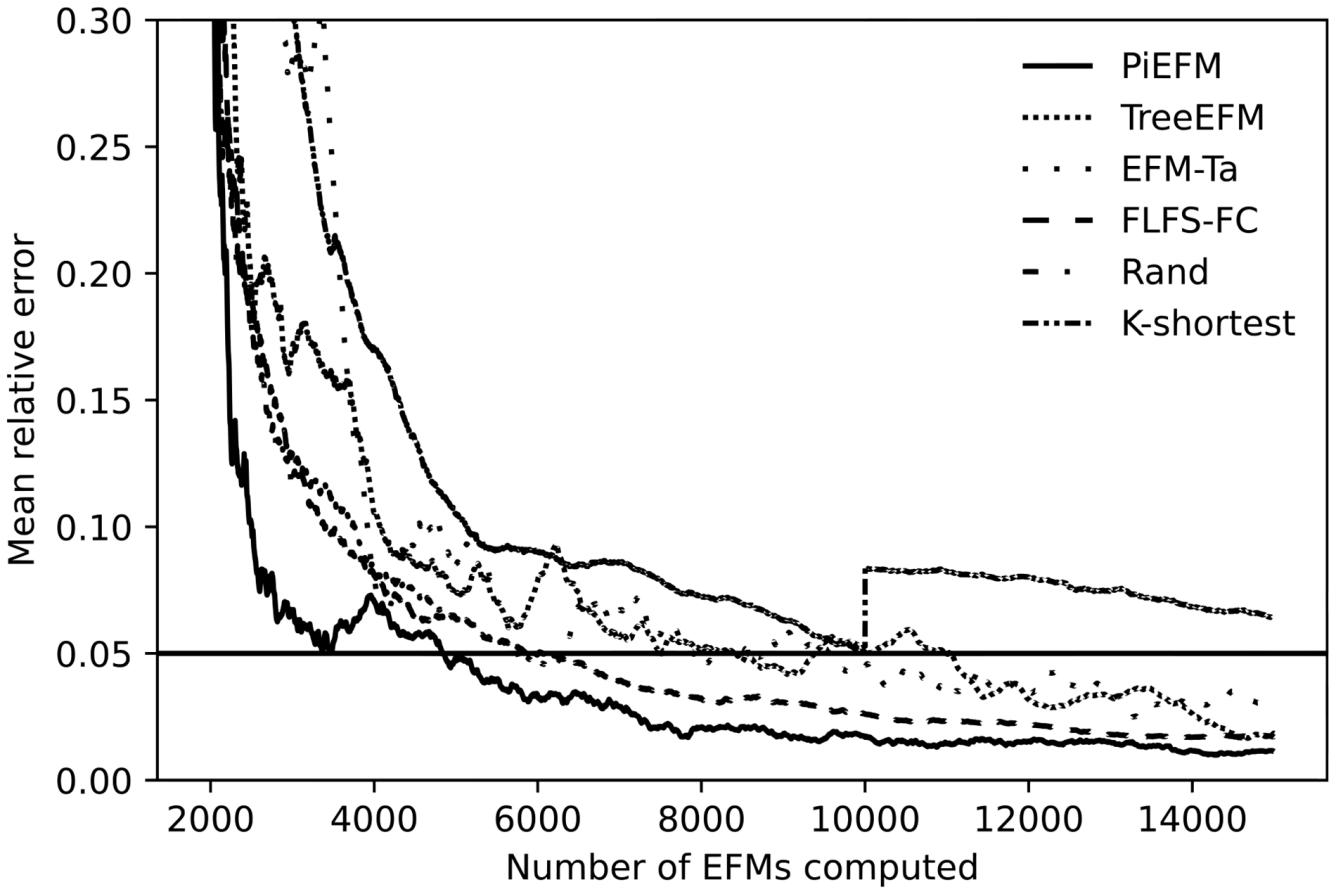
Evolution during the experiment of the mean relative error between the proportions of EFMs containing each reaction at two different moments *k* and *k−*2000 calculated on the computed EFMs using different methods in *core E.coli*.

In cases where the complete list of EFMs cannot be obtained (medium and large networks), the above study is replaced by stability. To do so, fix an offset *d* and compare the approximations to the proportions *p*(*r*) between different stages of the extraction method $p_{k}(r)$ and $p_{k-d}(r)$. If the method is correctly approximating the values of *p*(*r*) then the values of $p_{k}(r)$ and $p_{k-d}(r)$ should be very close. Figure 6 shows the evolution of the relative error between $p_{k}(r)$ and $p_{k-d}(r)$ for *d*** **=** **2000.

In this case, the horizontal line indicates when the mean relative error between $p_{k}(r)$ and $p_{k-d}(r)$ is less than 0.05. From these graphics, we can infer the moment the extraction method can stop as the number of obtained EFMs for which these mean relative errors are consistently below that line.

In this case, the best stability values correspond to TreeEFM, FLFS-FC, and PiEFM.

In this network, all non-blocked reactions are compatible with the Biomass reaction and are quickly detected by using any of the studied methods. So, we have not included this metric in this analysis.

Clearly, other metrics could have been included in this study. As an example, we have included a study of the evolution of the Jaccard distance (Jaccard 1912) between consecutive EFMs computed using different methods in the Supplementary Material.

### 4.2 EFM extraction methods in *iAF1260b*

In this second case study, the network *iAF1260b*, a genome-scale model for *E.coli (*Feist *et al.* 2010) that has 1668 metabolites and 2388 reactions, is used. This model has also been retrieved from BIGG Models using CobraPy.

In this case, the number of EFMs in the model is unknown, so our analysis is based on stability. We use those methods that have exhibited a good behavior in the *core E.coli* case, i.e. PiEFM, TreeEFM, EFM-Ta, and FLFS-FC.

As for treeEFM, we start by finding the reaction *GLCptspp* equivalent to the reaction *GLCpts* used in our previous model. We have put it as the target reaction and computed 200 000 EFMs in *iAF1260b*. However, it is observed that most of the 200 000 obtained EFMs do not contain the Biomass reaction. This behavior produces the generation of short EFMs, so we can be sure that a highly biased set E′ is produced this way. To avoid this problem, *BIOMASS_Ec_iAF1260_core_59p81M* (the biomass reaction of iAF1260b) has been chosen as a target.

PiEFM runs the same algorithm used in *core E.coli*, with a slight modification by computing around 4000 EFMs in the first step and applying the pivoting step to obtain 200 000 EFMs containing Biomass. We have also slightly modified EFM-Ta and EFLS-FC to produce only EFMs containing Biomass. To do so, we have used the positive constraint associated with the Biomass reaction as an additional constraint in all the optimization problems.

**Figure 7.**
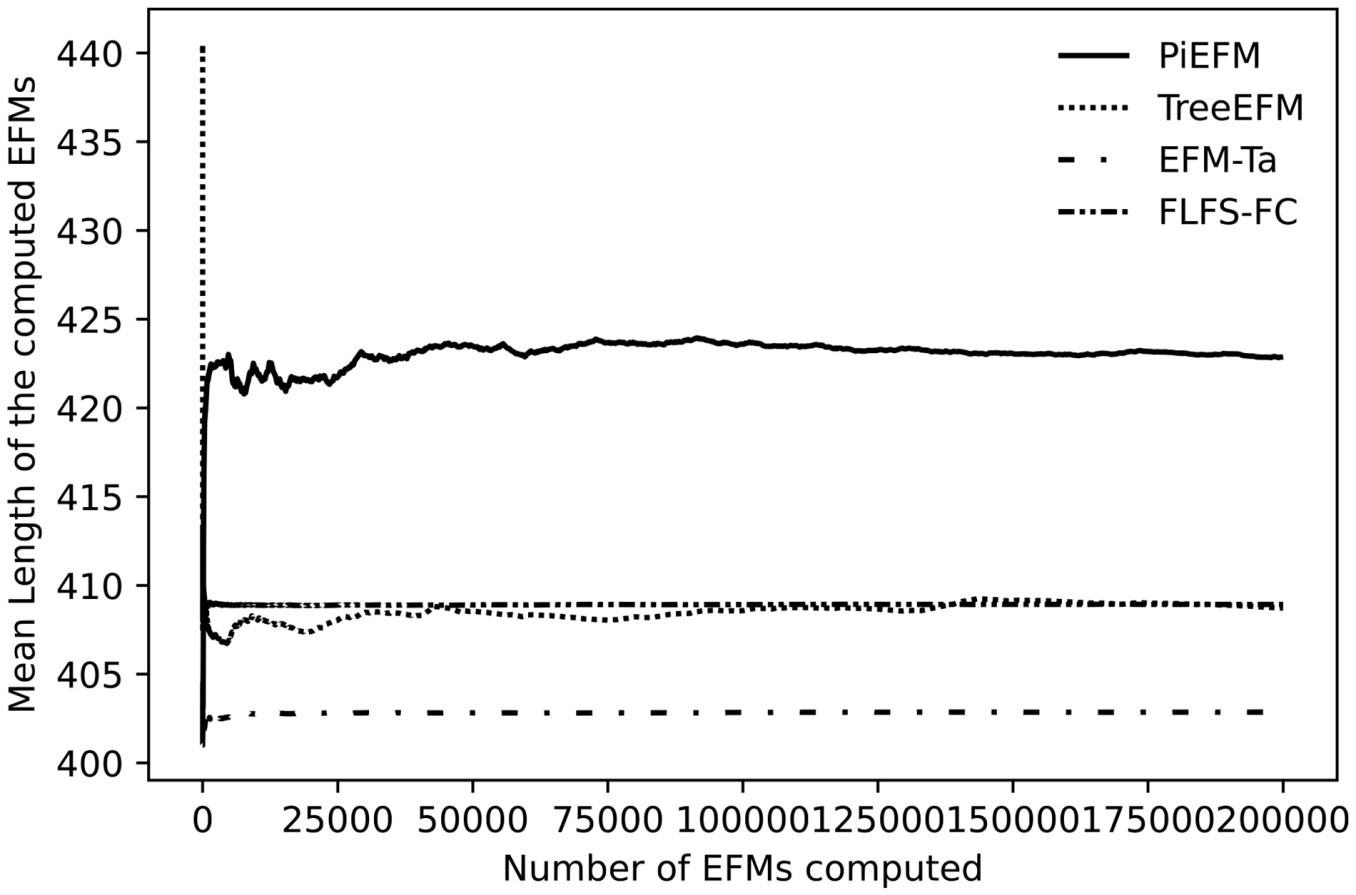
Evolution during the experiment of the mean length of those EFMs containing the Biomass reaction of different methods in *iAF1260b*.

Then the evolution of the mean length of the EFMs obtained using each methods has been analyzed. The results are shown in Fig. 7.

As in the model *core E.coli*, all methods tend to stabilize and PiEFM produces EFMs of higher lengths. As always, we cannot ensure that this is the correct mean length of the EFMs in *iAF1260b*, just that these are the mean lengths we expect from those methods.

**Figure 8.**
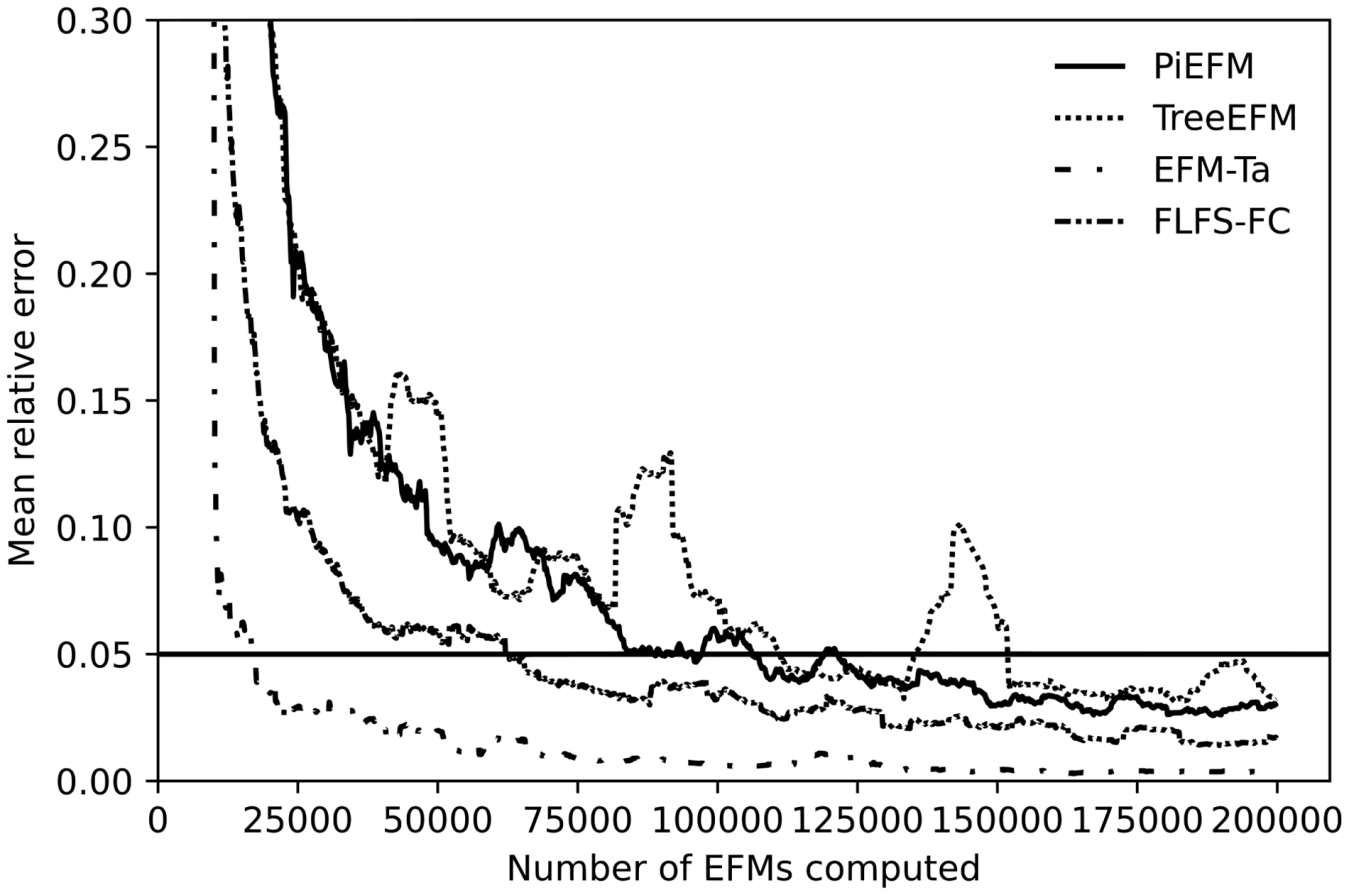
Evolution during the experiment of the mean relative differences between $p_{k}(r)$ and $p_{k-10000}(r)$, where *k* represents the number of EFMs computed so far in *iAF1260b*.

As for the proportion of EFMs that contains each reaction, we can compare the evolution of the mean relative error between the values obtained at each step and the final ones (or the corresponding correlation coefficients). As observed before, a possible way to do this is to measure the relation between the proportions at each step $p_{k}(r)$ and the ones obtained a certain number *d* of steps before, $p_{k-d}(r)$. We can see the evolution of these proportions in Fig. 8 (in this case, we have taken *d*** **=** **10 000).

The low value of these relative differences in the mean only ensures that our method obtains EFMs of the same kind (concerning the parameters under study) and that this is a necessary (but not sufficient) condition for our set of EFMs to be representative. However, we can be confident that we must continue the extraction process when we observe values over a certain threshold (0.05 in Fig. 8). PiEFM shows better stability than TreeEFM, so the extraction process could have been stopped earlier. But EFM-Ta and FLFS-FC show even better stability.

**Figure 9.**
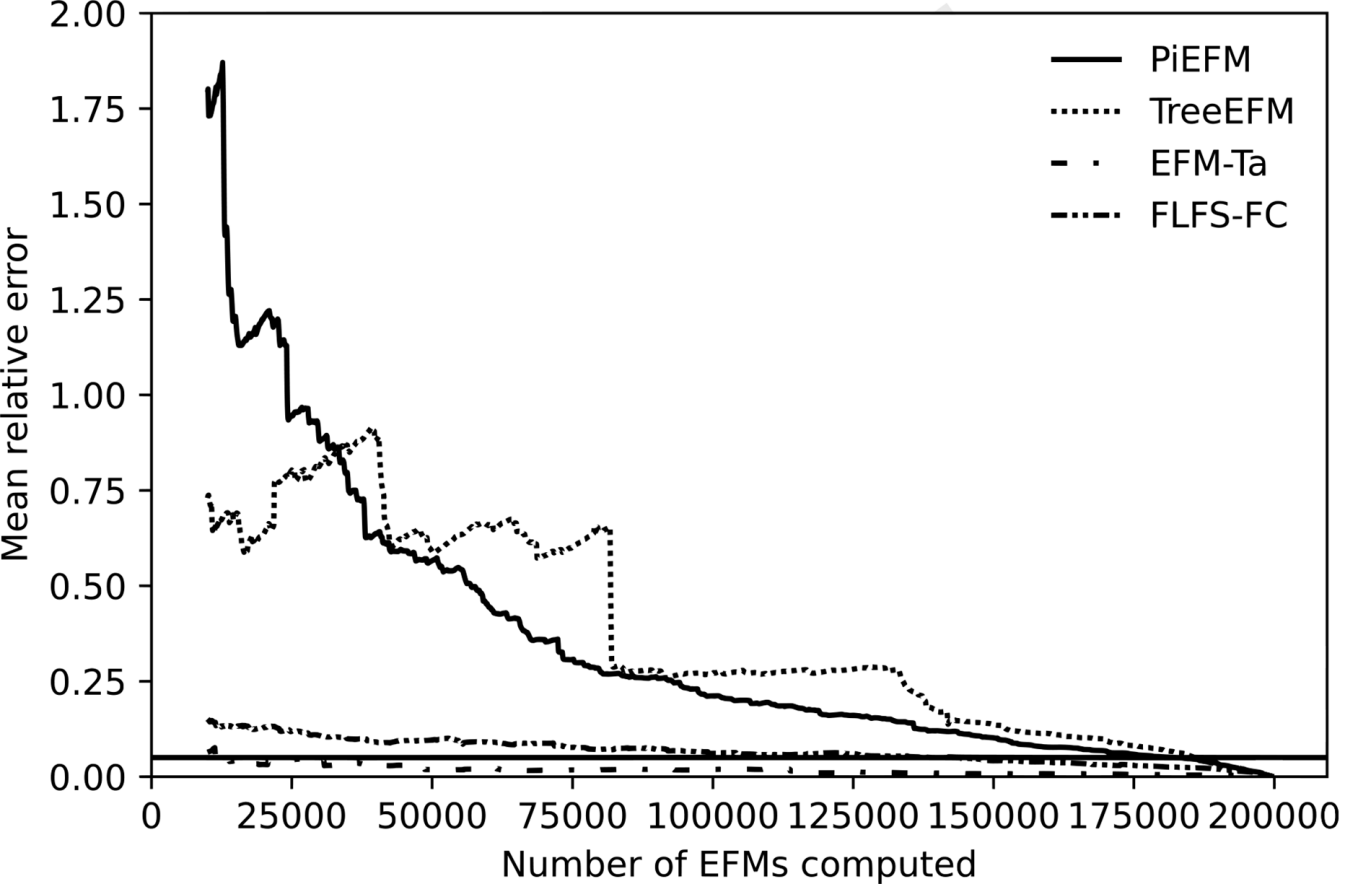
Evolution during the experiment of the mean relative differences between $p_{k}(r)$ and the final obtained ones $p_{200000}(r)$, where *k* represents the number of EFMs computed so far in *iAF1260b*.

The same conclusion can be seen again in Fig. 9 in which we obtain the mean relative differences between values $p_{k}(r)$ obtained during the extraction process (in each method) and the finally obtained ones.

**Figure 10.**
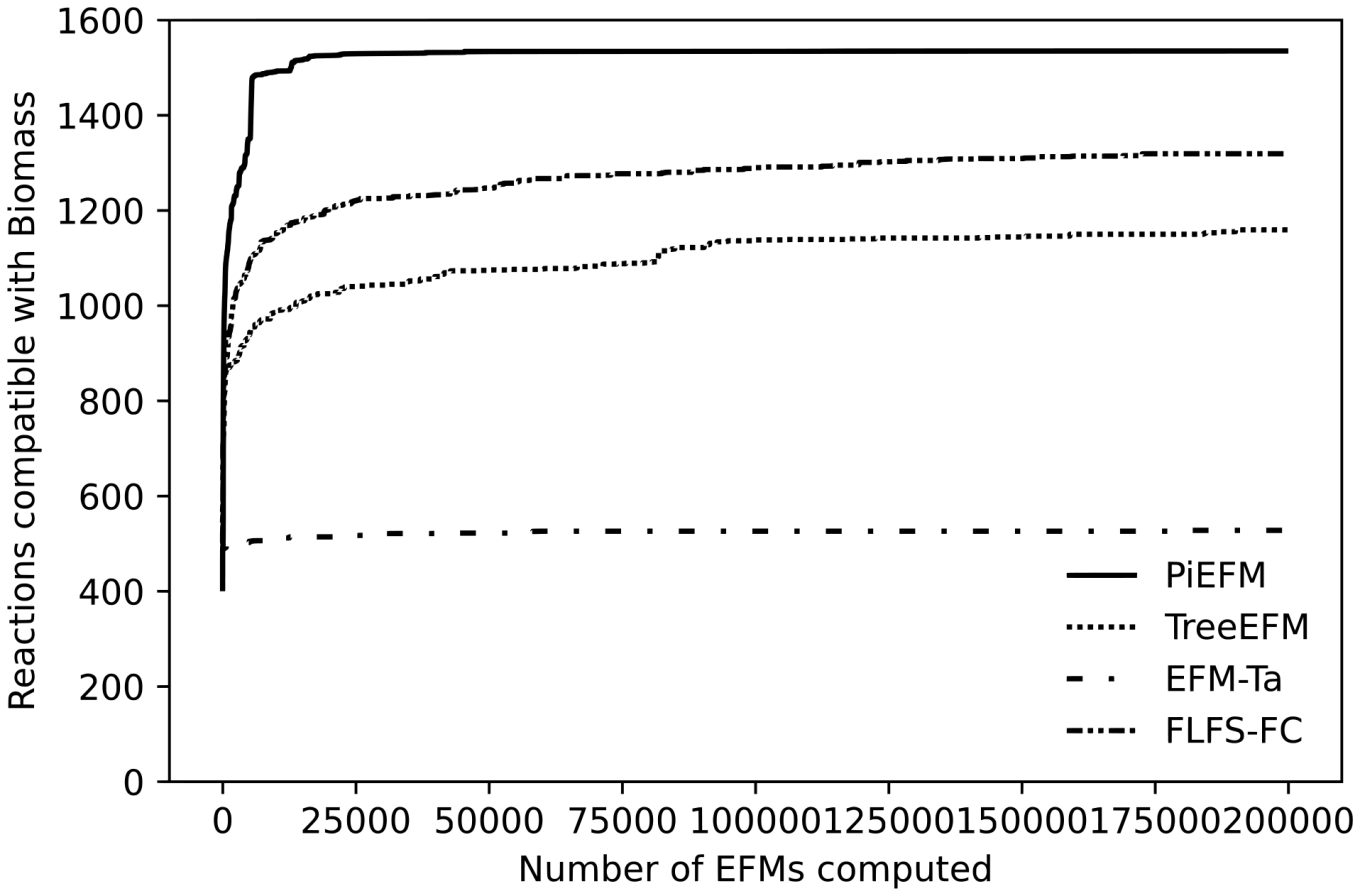
Evolution during the experiment of the number of reactions that are compatible with the Biomass reaction in *iAF1260b*.

As has been previously suggested, we can also use the number of pairs of compatible reactions to measure the variability of the EFMs obtained. In our case, all the computed EFMs contain the Biomass reaction as active. It is easy to approximate the number of reactions compatible with this reaction by just taking those reactions that appear in any of our EFMs. We have done so for our methods and the results can be viewed in Fig. 10.

We have found 1535 reactions compatible with the Biomass reaction using the 200 000 EFMs computed by PiEFM, 1159 using those computed by treeEFM (most of them, 1150 out of 1159, had also been obtained by PiEFM), 1329 using the EFMs calculated by EFM-Ta, and only 529 with the ones associated with FLFS-FC. That suggests that the EFMs computed by PiEFM exhibit more variability and that the stability observed while analyzing the FLFS-FC could be due to the similitude between the obtained EFMs.

## 5 Conclusions

EFMs are crucial in studying metabolic networks, but their cardinality is extremely large for genome-scale networks. This central problem can be faced with algorithms that only compute smaller subsets of EFMs, and these algorithms produce biased subsets.

This article proposes a methodology to study the quality of the subset of EFMs obtained by a certain (biased) algorithm. We have shown how several metrics can be used to study different biases that can (and surely will) appear.

We have also presented the concept of stability for a particular parameter and its relation to the representativeness of the method studied. Stability is crucial because it can be studied in cases where the whole set of EFMs is unavailable. Stability is also related to a central problem of these biased methods: where can we stop an EFM extraction process? We have shown a partial answer: the process must continue at least until all the relevant parameters are stable.

Finally, we have developed a new EFM extraction method, PiEFM, that can be seen as a mixture of biased and non-biased methods and shows good quality indicators.

We have used these tools to analyze the behavior of previously proposed methods in two case studies and show how these algorithms can be of different value while tackling various problems. In the first case study (*core E.coli*), we have shown that all the analyzed methods are biased, but not all have the same degree of bias, and that this can be detected using our proposed representativeness measures. In this case study, all the EFMs can be computed using non-biased methods, but it can be used as a test case to detect which methods exhibit better behavior.

In the second case (*iAF1260b*), the number of EFMs is unknown, so only biased methods can be used. We have selected those methods with good behavior in *core E.coli* and showed how to compare them using only the stability of parameters. We infer from the obtained results that PiEFM tends to be more stable (so probably less biased) than FLFS-FC, EFM-Ta, and treeEFM and exhibits a better variability in the set of extracted EFMs.

This methodology helps to analyze and improve future algorithms for the computation of subsets of EFMs in large networks.

## Supplementary Material

btad356_Supplementary_Data
