# Supplementary material for: On the representativeness and stability of a set of EFMs
Source: Bioinformatics. 2023 May 30;39(6):btad356. doi: 10.1093/bioinformatics/btad356 (PMC10264373; doi:10.1093/bioinformatics/btad356)
Supplement: btad356_Supplementary_Data [file btad356_supplementary_data.pdf]

Supplementary Materials

1 The role of the mean length of EFMs to study possible biases on an EFM extraction algorithm based on optimization methods

The majority of the evaluated methods are based on optimization techniques. In order to compute the different EFMs, they usually rely on minimizing ad-hoc linear functions with non-negative coefficients. Due to this behavior, they tend to produce short-length EFMs.

Figure 1 shows the histogram of lengths of the whole set of EFMs in *E. coli core*. As it can be noted, most EFM lengths are close to 49.

Figures 2, 3, 4, 5, and 6 show the comparison between the histogram of lengths of the whole set of EFMs versus the lengths of the EFMs computed using the different methods analyzed (all these histograms have been calculated based on the first 15000 EFMs obtained for each method).

The main problem is that EFMs with different lengths represent different network behaviors. This problem can be observed in Figures 7 and 8, representing the expected behaviour of EFMs of short and large lengths. In each figure, those active reactions in no EFM are drawn in red, while those always active are highlighted in green.

Observe, for example, that in all EFMs of short length, the reactions belonging to the Pentose Shunt metabolic block are always inactive, but the same reactions are almost always active in EFMs of great length. Similar differences can be observed in other blocks of the network.

This kind of 'families' of EFMs is the main reason for the non-unimodality of the histogram of lengths for the whole set of EFMs.

So, we think that the mean length of the computed EFMs can serve as a first metric to study biases associated with optimization methods.

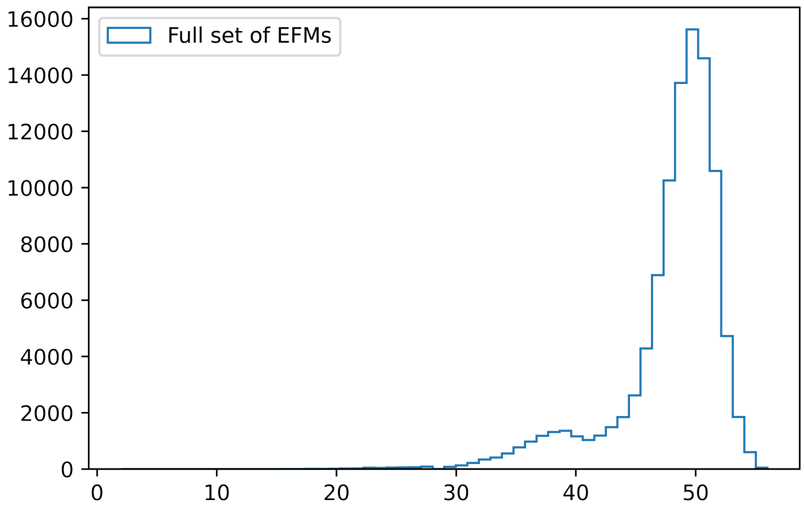

Figure 1: histogram of lengths of EFMs in *E. coli core*

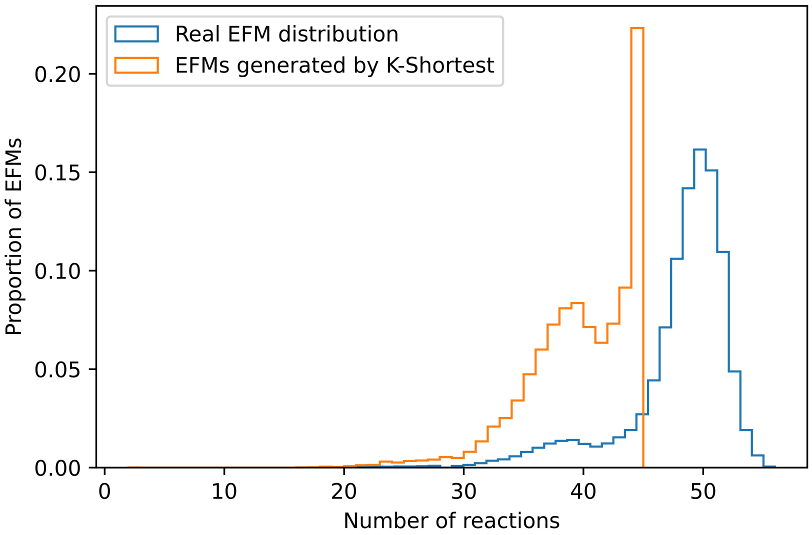

Figure 2: Comparison between the histogram of lengths of EFMs in *E. coli core* and those computed using the K-Shortest algorithm

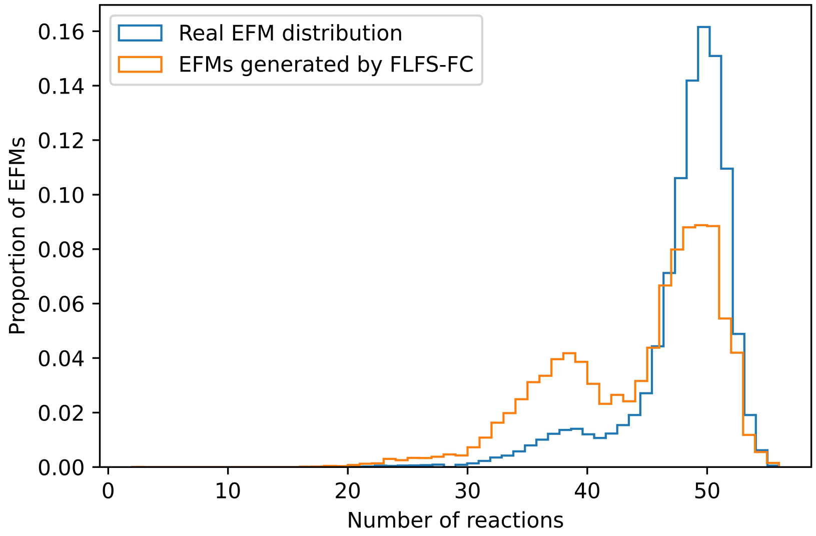

Figure 3: Comparison between the histogram of lengths of EFMs in *E. coli core* and those computed using the FLFS-FC algorithm

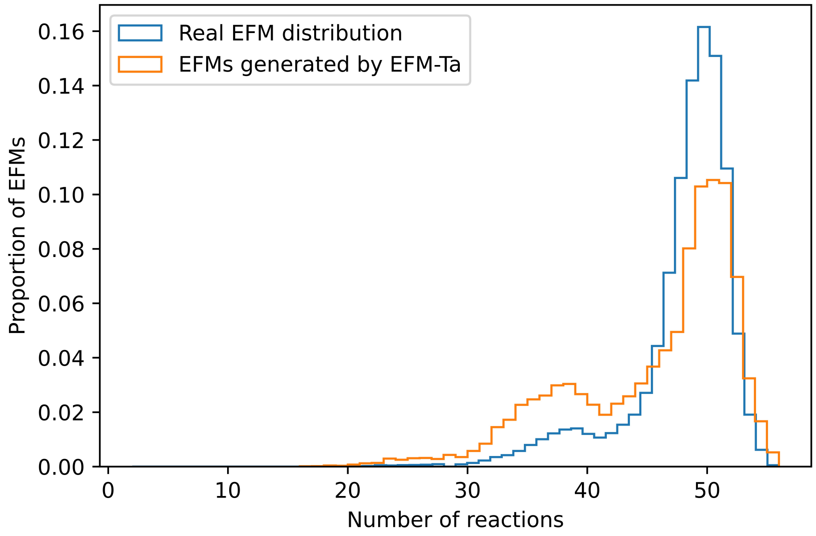

Figure 4: Comparison between the histogram of lengths of EFMs in *E. coli core* and those computed using the EFM-Ta algorithm

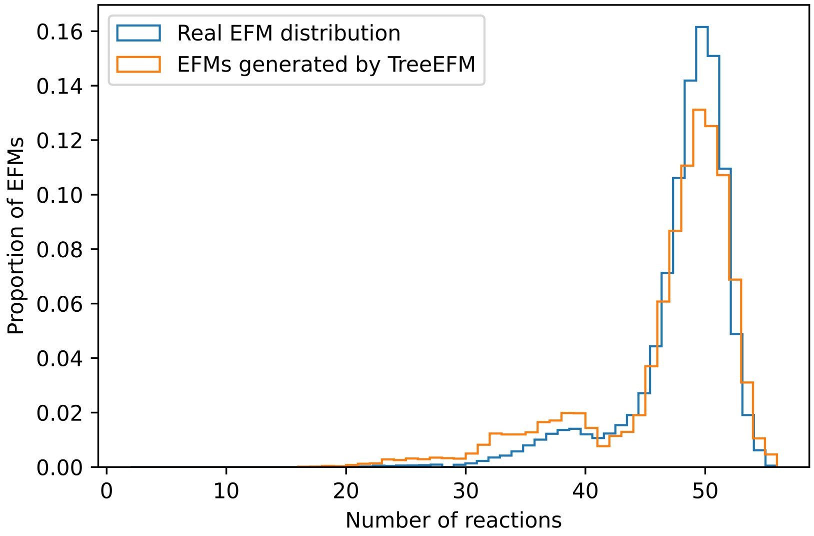

Figure 5: Comparison between the histogram of lengths of EFMs in *E. coli core* and those computed using the TreeEFM algorithm

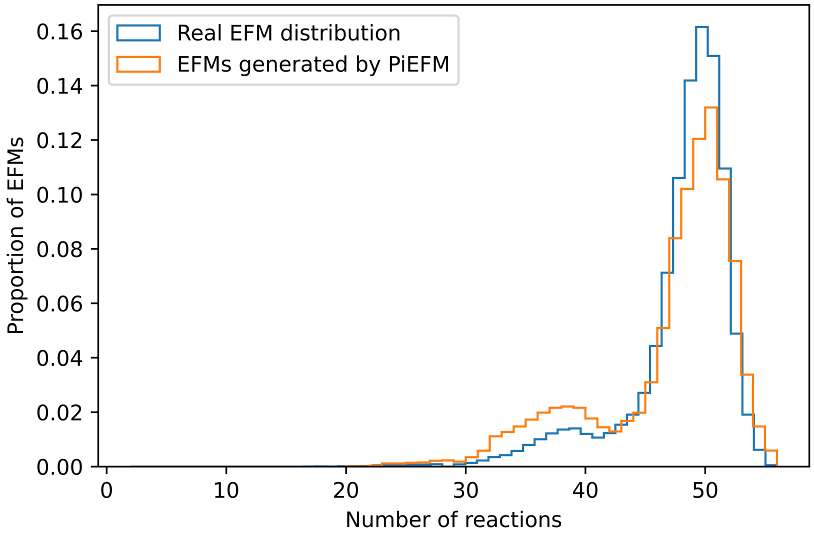

Figure 6: Comparison between the histogram of lengths of EFMs in *E. coli core* and those computed using the PiEFM algorithm

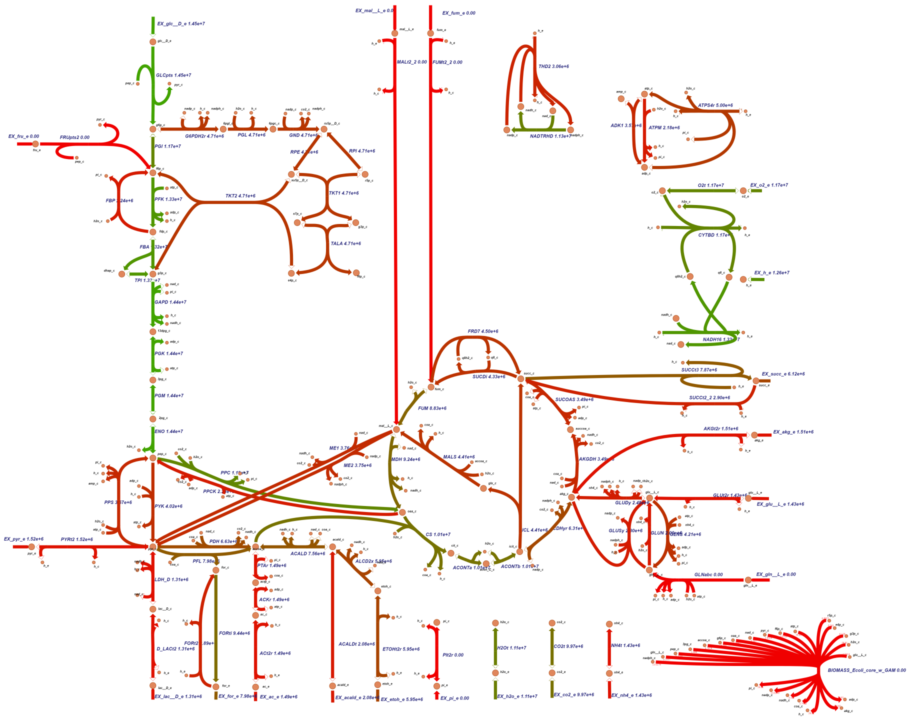

Figure 7: Common behaviour of those EFMs of *e. coli core* having a length less than 40. In this figure, those active reactions in no EFM are drawn in red, while those always active are highlighted in green.

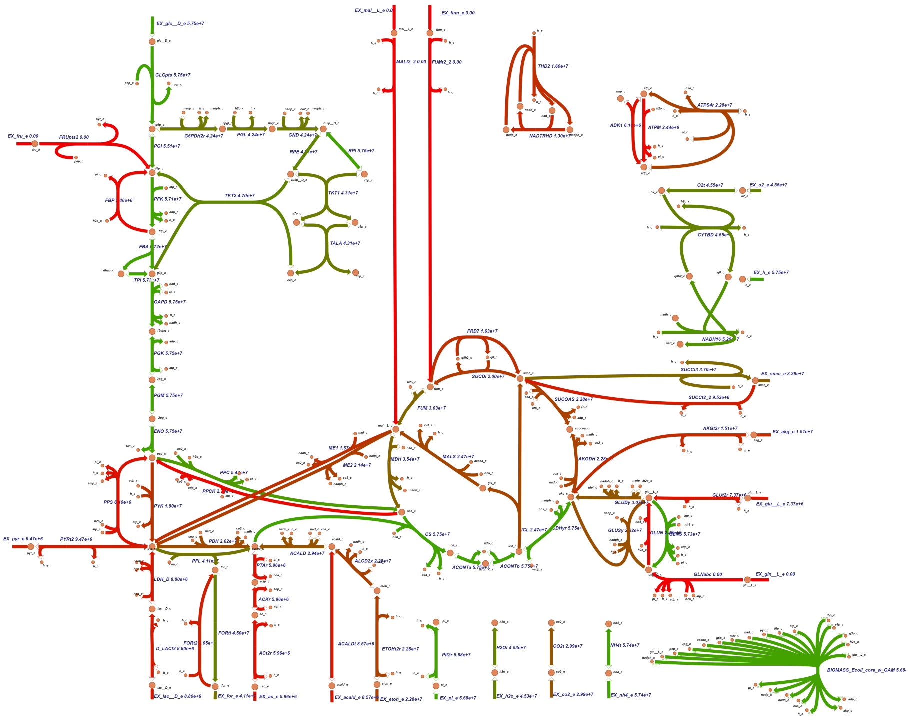

Figure 8: Common behaviour of those EFMs of *e. coli core* having a length between 47 and 51. In this figure, those active reactions in no EFM are drawn in red, while those always active are highlighted in green.

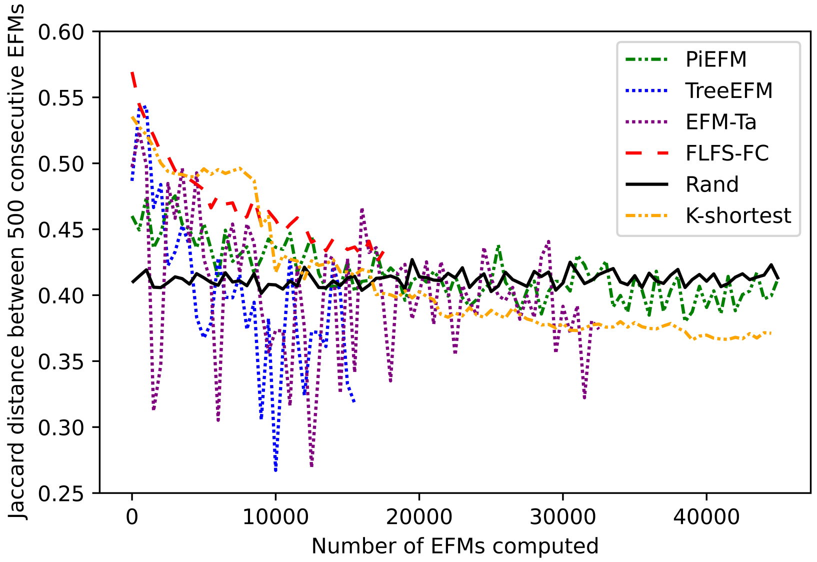

Figure 9: Evolution of the Jaccard distance between 500 consecutive EFMs computed using different methods

2 Other metrics for the study of the representativeness of a set of EFMs: the Jaccard distance

The Jaccard distance between two sets A and B is defined as $1 - \frac{|A \cap B|}{|A \cup B|}$. So it can be used to compare the different behaviour of sets of EFMs produced by different extraction methods.

As a case study, we have chosen a fixed size of $k = 500$ EFMs and studied the evolution of its Jaccard distance throughout the extraction process. Figure 9 shows the evolution of this mean Jaccard distance between 500 consecutive EFMs obtained using different algorithms. For comparison purposes, we have also included an 'ideal' purely random method consisting of randomly sampled sets of 500 EFMs. It can be observed that this metric is relatively stable while using random sampling (the solid black line), and its values are significantly different from the ones obtained by using the K-shortest algorithm (the orange dash-dotted line). We can again speak of the stability of the corresponding curves. Unstable curves tell us that the sets of EFMs obtained in different moments of the calculation behave differently, so they are less reliable than the more stable ones.

Several modifications can be performed in this definition. For example,

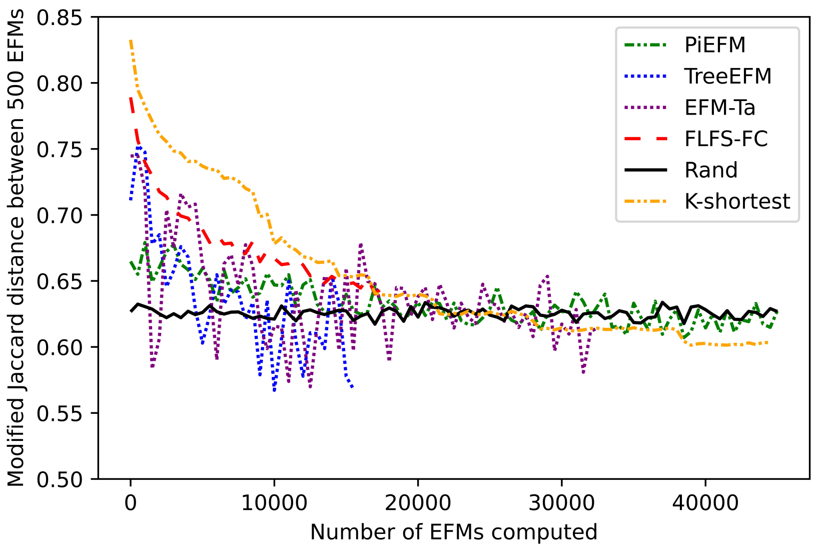

Figure 10: Evolution of the modified Jaccard distance between 500 consecutive EFMs computed using different methods

considering that all EFMs are subsets of the set of reactions, we can define a (modified) Jaccard distance as $1 - \frac{|A \cap B|}{|R|}$, where R = set of reactions of the network. The effect of this modification can be observed in Figure 10.

In this case, we obtained a slightly modified version of Figure 10. Again the lowest values are obtained by random sampling, and the most relevant feature seems to be the stability of the curve.

We can vary the smoothness of these curves by choosing different values of k, where higher values correspond to smoother curves. Figures 11, 12 and 13 shows the evolution of the modified Jaccard distance between k consecutive EFMs computed using different methods for $k = 1000$, 2000 and 5000.

3 Study of the efficiency of the PiEFM method in terms of time and memory consumption

In general, the most efficient algorithms in terms of memory usage are those based on optimization techniques. Algorithms in this family mainly use memory to store the computed EFMs and that needed by the solver while solving an optimization problem. PiEFM is efficient in terms of memory because it is far more efficient than those based on DDM and its efficiency is even slighly better than other based in LP techniques.

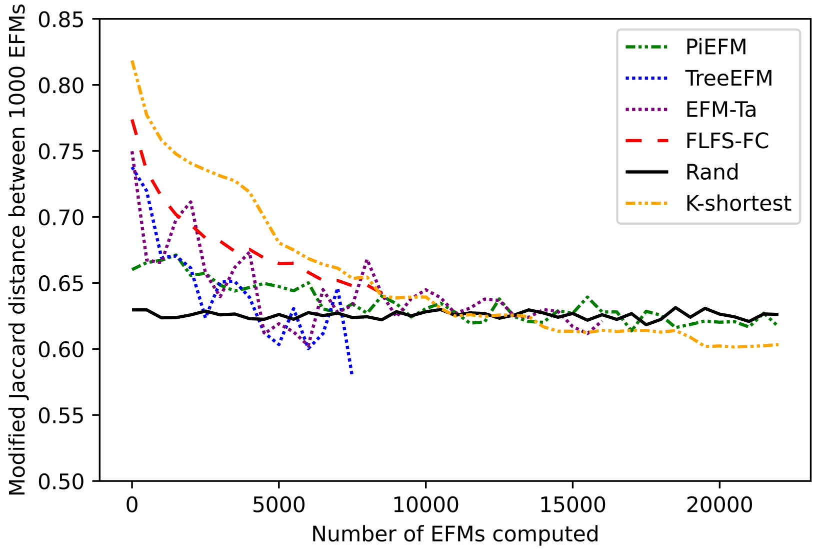

Figure 11: Evolution of the modified Jaccard distance between 1000 consecutive EFMs computed using different methods

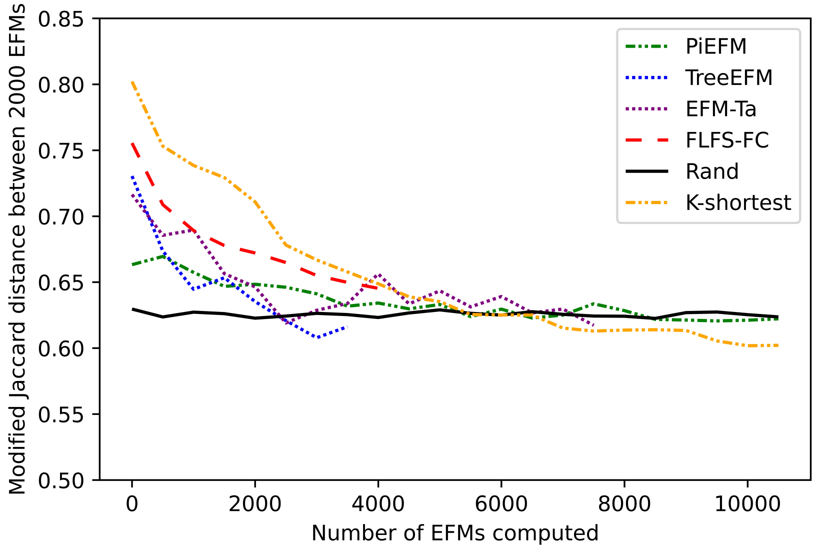

Figure 12: Evolution of the modified Jaccard distance between 2000 consecutive EFMs computed using different methods

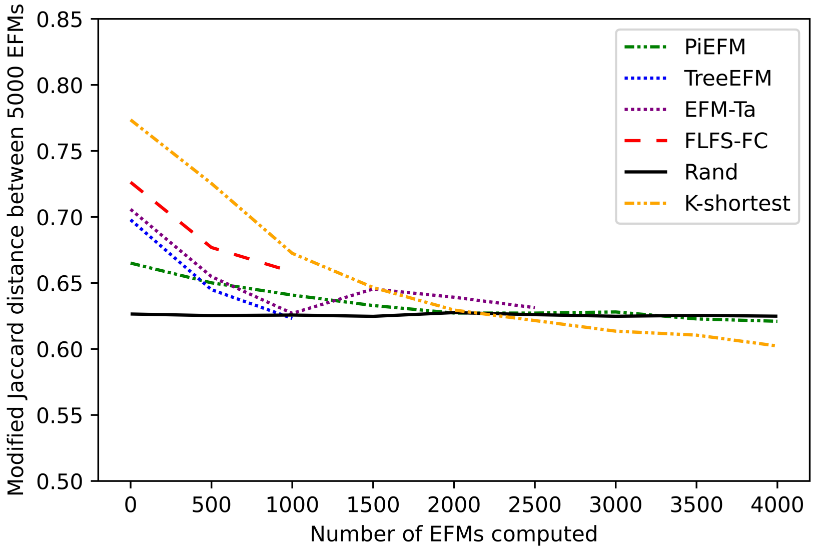

Figure 13: Evolution of the modified Jaccard distance between 5000 consecutive EFMs computed using different methods

PiEFM requires 199 MB of RAM memory to compute the first 10000 EFMs and most of the memory requirements are used in loading the required data and libraries, mainly CPLEX and NumPy (142 out of the 199 MB used, that is, approximately 71% of the memory requirements). The RAM needed increases to 272 MB to compute the first 100000 EFMs.

Regarding time execution, PiEFM needs less that 12 seconds to compute the first 50000 EFMs in *e. coli core* while, for example, EFM-Ta needs approximately one hour to perform the same task.

Figures 14 and 15 shows the memory usage and execution times for PiEFM while computing the first 100000 EFMs.

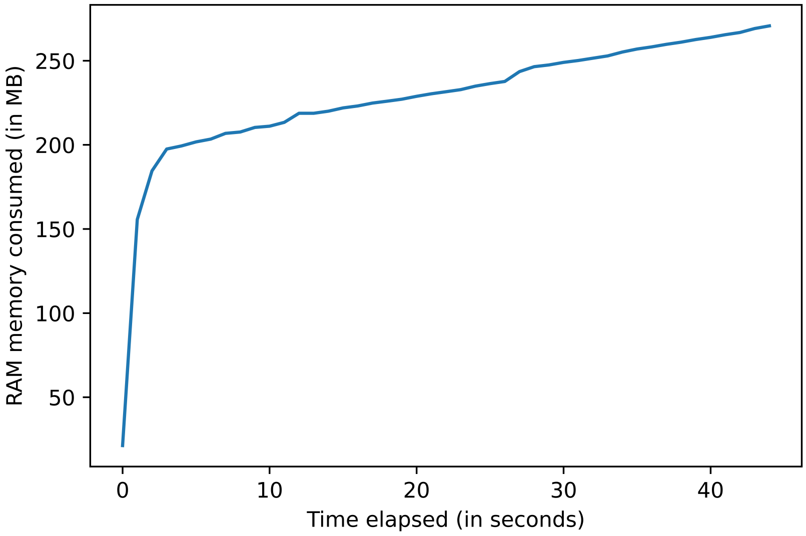

Figure 14: RAM memory used by PiEFM for the first 100000 EFMs in *e. coli core*

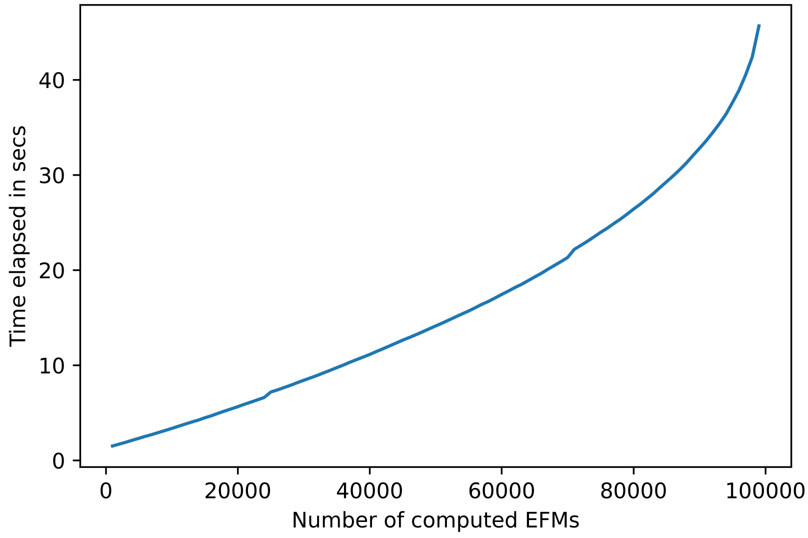

Figure 15: Time consumed by PiEFM for the first 100000 EFMs in *e. coli core*
